# Moderators of Food Insecurity and Diet Quality in Pairs of Mothers and Their Children

**DOI:** 10.3390/children9040472

**Published:** 2022-03-29

**Authors:** Christine Aggeli, Maria Patelida, Maria G. Grammatikopoulou, Ekaterini-Avrakomi Matzaridou, Marina Berdalli, Xenophon Theodoridis, Konstantinos Gkiouras, Angeliki Persynaki, Kyriaki Tsiroukidou, Theodore Dardavessis, Christos Tzimos, Dimitrios G. Goulis, Tonia Vassilakou

**Affiliations:** 1 Department of Medicine, Faculty of Health Sciences, Aristotle University of Thessaloniki, GR-56429 Thessaloniki, Greece; aggeli.christine@gmail.com (C.A.); xenophontheodoridis@gmail.com (X.T.); kostasgkiouras@hotmail.com (K.G.); 2 Department of Nutritional Sciences & Dietetics, Faculty of Health Sciences, International Hellenic University, Alexander Campus, GR-57400 Thessaloniki, Greece; patimary@yahoo.gr (M.P.); avroulam@gmail.com (E.-A.M.); marinamarios@hotmail.com (M.B.); agpersy@hotmail.com (A.P.); 3 3rd Department of Pediatrics, Hippokration General Hospital of Thessaloniki, Aristotle University of Thessaloniki, GR-54124 Thessaloniki, Greece; ktsiroukidou@gmail.com; 4 Laboratory of Hygiene, Social & Preventive Medicine and Medical Statistics, Faculty of Health Sciences, School of Medicine, Aristotle University of Thessaloniki, GR-56429 Thessaloniki, Greece; dardaves@auth.gr; 5 Northern Greece Statistics Directorate, Hellenic Statistical Authority, 218 Delfon Str., GR-54646 Thessaloniki, Greece; ctzimos@gmail.com; 6 Unit of Reproductive Endocrinology, 1st Department of Obstetrics and Gynecology, Faculty of Health Sciences, Medical School, Aristotle University of Thessaloniki, GR-54124 Thessaloniki, Greece; dgg@auth.gr; 7 Department of Public Health Policy, School of Public Health, University of West Attica, Athens University Campus, GR-11521 Athens, Greece

**Keywords:** dietary patterns, parents, adolescence, food security, diet diversity, single-parent family

## Abstract

Research has suggested that maternal diet and characteristics may influence the diet of offspring during childhood. The present cross-sectional study aimed to assess the influence of distinct maternal characteristics and the diet quality of mothers on the prevalence of household food insecurity (FI) and the diet quality of children. A total of 179 mother–child pairs were recruited from two primary schools in the metropolitan area of Thessaloniki. The children were aged between 10 and 12 years old. Diet quality was assessed as the level of adherence to the Mediterranean diet (MD), with the use of the KIDMED for the children and the MedDietScore for the mothers. The household FI and the social and demographic characteristics of the mothers were also recorded, and anthropometric measures of both the mothers and their children were collected. Approximately ¼ (26.3%) of the pairs reported some degree of FI, with a greater prevalence (64.7%) within single-mother families. Moreover, FI affected the level of maternal MD adherence (*p* = 0.011). On the other hand, FI was decreased in households with a greater maternal educational level (OR: 0.25; 95% CI: 0.10–0.63) and conjugal family status (OR: 0.15; 95% CI: 0.87–0.52). Maternal adherence to the MD was inversely related to the respective adherence of their offspring (OR: 0.93; 95% CI: 0.86–0.997), suggesting that during periods of financial constraints, maternal diet quality is compromised at the expense of affording a better diet for the minors in the family.

## 1. Introduction

Since the 2008 financial crisis, austerity measures and the rise in commodity prices have increased the prevalence of food insecurity (FI) in many European countries, including Greece [1,2,3]. By the end of 2007, the recorded price index of important food commodities, including staples, increased by 24%, with dairy products being pricier by 58% [4]. The observed increases in food prices had a widespread impact on the nutritional and health status of affected populations [4]. The World Health Organization (WHO) and the Group of Eight (G8) summit recognized the financial crisis as a threat to global health, ringing the alarm for the onset of health inequalities [5]. Diet diversity is negatively related to food prices, with simulations showing that the mean energy consumption per capita declined during the period 2006–2010, increasing the number of people who were food-insecure and at risk of being hungry [6]. Vulnerable populations, including women, older adults, and children, appear to be particularly affected [1,2,7,8,9], with family structure [10], financial burdens [11], and parental unemployment [12] affecting the prevalence of FI and reducing diet quality [11,13,14,15,16,17].

A large body of evidence suggests that children living in food-insecure households demonstrate a lower dietary diversity score (DDS) and diet quality, a reduced intake of key micronutrients, and an increased intake of energy-dense foods [16,18,19,20,21]. Individuals experiencing FI are more likely to consume energy-dense, nutritionally poor foods, in order to suppress the feeling of hunger [8,22,23]. Within families, however, it has been suggested that parents tend to shield their children from the negative health effects of FI by reducing their own diet quality while catering for the younger ones [19,24]. Moreover, we are aware that the quality of a mother’s diet is positively associated with her children’s dietary indicators [25,26], and that individual parental characteristics, such as education or employment status, may act as important effectors of a minor’s diet quality [27,28,29]. Furthermore, maternal dietary diversity is representative of the average household nutrient adequacy [30], with a low maternal diet diversity score being related to infant stunting [31] and child underweight [32].

The present cross-sectional study aimed to evaluate the relationship between maternal characteristics, maternal diet quality, household FI, and the quality of children’s diets in a sample of mother–child pairs.

## 2. Materials and Methods

### 2.1. Sample Collection

During the year 2017, a total of 179 pairs of mothers and their children (10–12 years old) attending two public primary schools in Thessaloniki (from the areas of Neapoli and Ampelokipi), Greece, were recruited.

Inclusion criteria were: (1) adolescent children, aged 10–12 years old (students in the two senior primary school classes); (2) willing to participate; and (3) with an adult mother living the in same household as them. Exclusion criteria were: (1) children of younger ages; (2) no provision of parental/guardian participation consent; (3) having adolescent mothers; and (4) having mothers who were not residing with them in the same household. Sample characteristics are presented in Table 1.

### 2.2. Weight Status and Anthropometry

In children, body weight was measured during morning hours using a digital Seca 874 scale (Seca GmbH and Co., Hamburg, Germany) [33]. Height of children was measured with a portable stadiometer (Seca 214, Seca GmbH and Co., Hamburg, Germany). Maternal weight and height were reported.

Waist circumference was measured at the iliac crest [34,35], and waist-to-height ratio (WHtR) was also calculated.

Body mass index (BMI) was calculated for each participant (mother or child) as the body mass (kg) divided by height squared (m^2^). For the assessment of body weight status, the International Obesity Task Force (IOTF) [36] criteria were employed for the children, and the World Health Organization (WHO) cutoffs for BMI were used for the mothers [33].

### 2.3. Diet Quality

Diet quality was evaluated as the level of adherence to the Mediterranean diet (MD). For children, the Mediterranean Diet Quality index for children and adolescents (KIDMED) questionnaire was used [37]. The KIDMED consists of 16 food-frequency questions based on the main components of the MD. Total KIDMED scores range between 0 and 12, with greater scores being indicative of greater MD adherence. Scores >8 indicate optimal adherence and diet quality, scores ranging between 4 and 7 suggest moderate adherence, and scores <3 indicate the adoption of a diet in need of improvement.

Similarly, for the women, the Mediterranean Diet Score (MedDietScore) [38] was employed. Total scores range between 0 and 55, with greater scores suggesting better diet quality on the basis of the MD principles. Maternal diet quality was categorized as good when the MedDietScore exceeded 45 points, average at 23 < MedDietScore < 45, and low when the calculated score was below 23 points in total.

### 2.4. Dietary Intake

Dietary intake was assessed using 24 h recalls, with the assistance of experienced dietitians (M.P., E.-A.M., and M.B.). Records were analyzed using Food Processor software (ESHA Research, Salem, Oregon), and the daily intake of energy, protein, and selected micronutrients was calculated for each participant.

### 2.5. Diet Diversity

The household diet diversity score (HDDS) [39] was employed to evaluate variety in the dietary intake of mothers and their children. The questionnaire consists of 12 binary (yes/no) questions, each receiving 0/1 points, evaluating the consumption of different food groups. Scores closer to 12 (maximum score) are indicative of greater diet diversity. The HDDS has been previously used in the Greek population [2].

### 2.6. Household FI

Household FI was evaluated using the Household Food Insecurity Access Scale (HFIAS) [39]. The questionnaire consists of 9 questions evaluating uncertainty and stress regarding food access, food quality, and food quantity during the past month. The total HFIAS score ranges between 0 and 27, with greater scores being indicative of greater FI. The questionnaire has been previously used in the Greek population [1,2].

### 2.7. Statistical Analyses

Normality in the distribution of the variables was assessed through graphs and the Kolmogorov–Smirnoff test. Independent sample *t*-tests were used to identify differences in normally distributed variables, and the Mann–Whitney U test for non-normally distributed data. Data are presented as means ± standard deviation (SD) (normally distributed data), or as medians and their respective interquartile ranges (IQR) for non-normally distributed data. McNemar–Bowker test was used to assess differences between nominal parameters. Spearman’s correlation coefficient assessed the correlation between children’s KIDMED and mothers’ MEDDIET scores.

Univariate logistic regressions were performed to identify the association between children’s MD adherence and FI (dependent variables) and each independent variable. Children’s MD adherence was coded as moderate/greater MD adherence versus lower MD adherence (reference group), while FI categories included any level of FI (low, moderate, or severe) vs. food security (reference category). In the aforementioned regressions, maternal MD adherence was the main variable of interest, and the rest of the independent variables can be seen as covariates. For the multivariate (ML) models, only those variables with a *p*-value < 0.200 were included. Similarly, the diet quality of the children (dependent) was also assessed through ML analysis.

Data were analyzed using Τhe Jamovi project (version 1.2.27.0) [40] and PASW Statistics 21.0 (IBM SPSS Inc., Hong Kong, China).

## 3. Results

### 3.1. Weight Status

The majority of mothers were normoweight (57.9%) (Table 2), with 31.4% of the mothers being classified as overweight, and the remaining 10.7% as obese. Likewise, the majority of children were normoweight (64%), 26.4% demonstrated an excess in body weight, and the remaining 9.6% were classified as obese. However, based on the McNemar–Bowker test, no statistical association was observed between the weight statuses of mothers and children.

### 3.2. MD Adherence

Only one of the mothers demonstrated optimal MD adherence (Table 3). The majority (98.9%) exhibited moderate adherence to the MD. On the other hand, the majority of children (59.8%) followed a diet of moderate quality, 25.7% adhered to a diet of optimal quality, and the remaining 14.5% demonstrated low diet quality.

There was a weak negative correlation between children’s KIDMED and mothers’ MEDDIET continuous scores, as tested by Spearman’s correlation coefficient (*r* = −0.101, *p* = 0.181). When children’s diet quality was explained through logistic regression, it was revealed that maternal MD adherence was a negative effector of a child’s adherence to the MD (Table 4).

### 3.3. Household FI

The HFIAS revealed that the majority of the mother–child pairs (74%) were food secure. On the other hand, 26% reported some degree of FI, with 11% experiencing mild FI, 8% living in moderate FI, and the remaining reporting severe FI. Between food secure and insecure households, mothers residing in the latter exhibited lower MD adherence (Table 5), but no other differences were noted regarding the characteristics of participant pairs (age, BMI, etc.), their dietary intake, or their diet diversity.

The prevalence of FI within single-parent families was 64.7%. As a result, single-parent families were more frequently encountered in the food-insecure households as compared to the food-secure ones (23.4% vs. 4.55%, respectively, *p* ≤ 0.001). No differences were noted in the prevalence of maternal or child overweight and obesity between food-secure and -insecure households or between single- and two-parent families (data not shown).

The ML model revealed that higher maternal education appeared to act protectively against the onset of FI, whereas having a single-parent family, with mothers being the primary caretakers, increased the odds of food insecurity vs. food security (Table 6).

## 4. Discussion

The present study indicates that 26.3% of the households with an adolescent aged between 10 and 12 years old experience some degree of FI. This prevalence is even greater (64.7%) within single-parent families with the mothers as the primary caregivers. Furthermore, the results suggest that household FI is dependent on maternal education and single-parent family status with mothers being the primary caregivers. Moreover, an early adolescent’s diet quality appears to be inversely related to the diet quality of the child’s mother. No differences were noted in the weight status of participants or their dietary intake between food-secure and -insecure households.

Previous research in Canada suggested that the children of food-insecure mothers tend to consume more unhealthy foods and demonstrate a reduced consumption of fruits and vegetables [20]. In the present study, FI was not an effector of a child’s diet quality; however, maternal diet quality was inversely associated with children’s adherence to the MD. According to a recent Spanish study [41] assessing the diet quality of mother–child (8–10 years old) pairs, the maternal intake of vegetables, fish, fruits, legumes, pasta/rice, dairy products, nuts, and baked goods was positively related with the corresponding child behaviors. Similarly, in Norway, maternal diet quality was the strongest prospective predictor of a child’s respective MD adherence at 3 and 8 years of age [42]. Similarly, research in Australia suggested that the maternal postnatal diet was more strongly associated with a child’s diet quality and fruit and vegetable variety compared to the diet adopted during the gestational period [43]. This indicates that children may also act mimetically, copying their mothers’ habits. In less-affluent countries, however, this pattern is slightly altered, but still valid. In rural Timor-Leste, mothers appear to adhere to a strikingly poor diet, low in animal-source foods, while providing more animal-source foods to their children [25]. Nevertheless, maternal dietary quality still explains a child’s diet in Timor-Leste [25] and Ethiopia [29]. In the present study, the inverse relationship between maternal and children’s diet quality is in contrast to the aforementioned examples. However, research has suggested that when experiencing financial difficulties, mothers tend to lower their diet quality in order to afford a good diet for their offspring [19,24]. Thus, it is likely that a tight financial situation might have been the driver of this behavior. Recently, another group of researchers also noted similar behaviors in Greece [44], with the parents reducing the quality of their own diet in an effort to secure a better diet for their children.

Research in adults has associated FI with reduced MD adherence [2,11,45] and low diet quality [13,14,15,19,46,47,48,49,50,51]. Among food insecure adolescents, those living in Lebanon exhibit a lower adherence to the MD [52,53], and in Taiwan they exhibit a diet of lower diet diversity [16]. In the U.S. [19], children with FI tend to have a lower intake than recommended for many important micronutrients, and in Canada, reduced diet quality is observed in food-insecure children, paired with a greater energy intake from ultra-processed foods [48]. However, the number of studies conducted on children and adolescents is limited; thus, it is difficult to discern if household FI has a significant effect on the diet quality of adolescents living in more affluent countries [18]. According to a meta-analysis [19] pooling a sample of U.S. adults and children, although FI is associated with a lower diet quality in the former, the children’s results appear less consistent.

Underemployment contributes to the defining characteristics of FI, namely economic strain, financial vulnerability, and poverty. A plethora of studies have shown that unemployment [54,55,56] and underemployment [57] are both associated with an increased risk of FI. In Quebec, food-insecure individuals are more likely to perform multiple jobs to reach a viable income and report higher job stress [57]. Moreover, gaining full-time employment status has been shown to decrease the severity of FI among low-income Canadian adults [58].

In the present study, the risk of FI was higher among single-mother families, whereas a greater maternal educational attainment reduced the odds of FI. Inevitably, when only one member of the family is bringing money into the household, the risk of FI increases [57]. Thus, research from Canada, Australia, and the U.S. agrees in the fact that FI is greater in single-mother households [57,59,60]. Herein, the prevalence of FI tripled in single-mother homes. In the U.S., 27.8% of households headed by a single woman were food-insecure during the year 2018, whereas in the present study, the respective prevalence was approximately triple. Nevertheless, is should be noted that the sample was not representative of the single mothers residing in the country, while in parallel, it is well-known that the results of the 2008 economic crisis were direr in Greece compared to other countries.

In the U.S., a 1% increase in annual inflation was associated with a 0.5% increase in the prevalence of FI [61], indicating the direct relationship between food prices and the incidence of FI. Thus, maternal diet quality and, by inference, FI are closely related to the SES and the level of educational attainment of mothers [62,63]. Moreover, FI appears to affect diet quality through the employment of specific food-shopping practices, driven mainly by an effort to reduce costs for food [15,64]. As a result, individuals with FI often select energy-dense foods of low micronutrient quality [15] and tend to make fewer shopping trips, relying mainly on nonfresh produce [65]. Low maternal education is associated with lower levels of nutrition knowledge, and this may affect household FI, through the inability or reduced ability to manage food resources. As a result, several lines of evidence have associated maternal education [63,66] and nutrition knowledge with household FI [67,68]. On the other hand, a greater ability and self-confidence in managing food resources in the household is associated with a lower risk of FI [69]. Thus, interventions aiming to increase maternal nutritional knowledge can also improve household FI [70,71].

The limitations of the present study include the relatively small sample and the lack of a representative sampling strategy. Nevertheless, this effort constitutes the first of its kind in the country, using mother–child pairs and aiming to evaluate diet quality. Previous research in Greece has revealed a similar prevalence of overweight and obesity among adolescents as well as a similar diet quality [72,73], indicating that the present sample was rather representative of the child population. 

## 5. Conclusions

Even marginal FI has been positively associated with adverse health outcomes [74], and the need to break the vicious cycle of life-long FI is evident. The present findings indicate that, irrespective of the general financial strain the Greeks are enduring, single-mother homes are particularly affected and require multidisciplinary state actions to alleviate the prevalence of FI and improve diet quality among children. Furthermore, the findings suggest that during periods of financial constraints, maternal diet quality is compromised at the expense of affording a better diet for the minors in the family.

## Figures and Tables

**Table 1:** Characteristics of the participating mother–child pairs (N = 179).

Child sex (boy/girl) (*n*)	92/87
Child age (years)	11.0 ± 0.9
Maternal age (years)	42.0 ± 4.6
Single-parent family (yes/no)	17/162
Maternal employment status (maternal leave/unemployed/employed part-time/employed full-time) (*n*)	8/44/101/26
Maternal educational level (primary school/secondary school/lyceum/university/postgraduate)	44/21/47/52/15

**Table 2:** Pairs of mothers and children in each weight status category (%, *n*).

	Mothers (N = 178) *				
Children (N = 178) *	Normoweight ^†^	Overweight ^†^	Obese ^†^	Total (Children)	*p*
	*n* (%)	*n* (%)	*n* (%)		
Normoweight ^‡^	41.6% (*n* = 74)	18% (*n* = 32)	3.9% (*n* = 8)	64% (*n* = 114)	0.624 ^¥^
Overweight ^‡^	12.9% (*n* = 23)	4.5% (*n* = 18)	3.4% (*n* = 6)	26.4% (*n* = 47)	
Obese ^‡^	3.4% (*n* = 6)	3.4% (*n* = 6)	2.8% (*n* = 5)	9.6% (*n* = 17)	
Total (mothers)	57.9% (*n* = 103)	31.4% (*n* = 56)	10.7% (*n* = 19)	100% (*n* = 178)	

* Data were missing for one participant; ^†^ according to the World Health Organization criteria [33]; ^‡^ according to the World Obesity criteria [36]. ^¥^ refers to the McNemar–Bowker test.

**Table 3:** Pairs of mothers and children in each MD adherence category (%, *n*).

	Mothers (N = 179)			
Children (N = 179)	Low MD Adherence ^†^	Moderate MD Adherence ^†^	Optimal MD Adherence ^†^	Total (Children)
Low MD adherence ^‡^	0% (*n* = 0)	14.5% (*n* = 26)	0.0% (*n* = 0)	14.5% (*n* = 26)
Moderate MD adherence ^‡^	0.6% (*n* = 1)	58.7% (*n* = 105)	0.6% (*n* = 1)	59.8% (*n* = 107)
Optimal MD adherence ^‡^	0% (*n* = 0)	25.7% (*n* = 46)	0.0% (*n* = 0)	25.7% (*n* = 46)
Total (mothers)	0.6% (*n* = 1)	98.9% (*n* = 117)	0.6% (*n* = 1)	100% (*n* = 179)

KIDMED, Mediterranean Diet Quality Index for children and adolescents [37]; MD, Mediterranean diet; MedDietScore, Mediterranean Diet Score [38]; ^†^ based on the MedDietScore [38]; ^‡^ based on the KIDMED [37].

**Table 4:** Univariate and multivariate logistic regression models explaining children’s MD adherence.

Variables	Univariate Analysis			Multivariate Analysis		
	OR	95% CI	*p*	OR	95% CI	*p*
Being FI (vs. food-secure)	1.33	0.63 to 2.78	0.456			
Single-parent family (vs. non-single-parent family)	1.14	0.35 to 3.68	0.830			
Maternal MD adherence ^†^ (continuous)	0.93	0.87 to 1.00	0.038	0.93	0.86 to 0.997	0.041
Maternal ΒΜΙ (continuous)	0.95	0.88 to 1.03	0.188	0.94	0.86 to 1.01	0.100
Employed (vs. unemployed)	0.90	0.42 to 1.94	0.783	0.69	0.32 to 1.47	0.333
Tertiary education (vs. lower educational level)	0.58	0.28 to 1.20	0.139			

BMI, body mass index; CI, confidence intervals; FI, food insecure [39]; MD, Mediterranean diet; OR, odds ratio; ^†^ based on the MedDietScore [38]. Note: the dependent variable was maternal high/moderate MD adherence vs. low MD adherence (reference group).

**Table 5:** Differences in maternal and child characteristics between food-secure and -insecure households (means ± SD, or medians and their respective IQR).

Characteristics of Participants		Food Secure (*n* = 132)	Food Insecure (*n* = 47)	*p*
Child characteristics	Age (years)	10.9 ± 0.9	11.1 ± 0.9	NS
	BMI (kg/m^2^)	20.0 ± 3.9	20.6 ± 4.7	NS
	WHtR	0.47 ± 0.05	0.47 ± 0.06	NS
	KIDMED	6.2 ± 2.4	6.0 ± 2.8	NS
	HDDS	7.4 ± 1.8	6.96 ± 1.65	NS
	Energy intake (kcal/day)	1408 (1155, 1605)	1270 (1086, 1688)	NS
	Protein intake (g/kg of BW/day)	1.22 (0.9, 1.61)	1.17 (0.93, 1.48)	NS
	Trans fat intake (g/day)	0.64 (0.48, 0.91)	0.56 (0.47, 0.77)	NS
Maternal characteristics	Age (years)	42.1 ± 4.3	41.9 ± 5.4	NS
	BMI (kg/m^2^)	25.2 ± 5.0	24.0 ± 3.8	NS
	MedDietScore	34.3 ± 5.0	32.2 ± 4.6	0.011
	HDDS	7.85 ± 1.9	7.87 ± 2.45	NS
	Energy intake (kcal/day)	1217 (958, 1436)	1255 (982, 1410)	NS
	Protein intake (g/kg of BW/day)	0.75 (0.56, 0.95)	0.72 (0.56, 0.84)	NS

BMI, body mass index; BW, body weight; HDDS, household diet diversity score [39]; IQR, interquartile range; KIDMED, Mediterranean Diet Quality Index for children and adolescents [37]; MedDietScore, Mediterranean Diet Score [38]; NS, not significant; SD, standard deviation; WHtR, waist-to-height ratio.

**Table 6:** Univariate and multivariate logistic regression models explaining household FI.

Variables	Univariate Analysis			Multivariate Analysis		
	OR	95% CI	*p*	OR	95% CI	*p*
Maternal BMI (continuous)	0.95	0.87 to 1.03	0.174	0.92	0.84 to 1.00	0.917
Conjugal family (vs. single-parent family)	0.16	0.05 to 0.45	<0.001	0.15	0.87 to 0.52	0.003
Maternal MD adherence ^†^ (continuous)	0.92	0.85 to 0.98	0.013	0.94	0.87 to 1.02	0.149
Higher maternal education (vs. lower educational level)	0.21	0.09 to 0.50	<0.001	0.25	0.10 to 0.63	0.003
Maternal employment status (vs. unemployment)	0.80	0.38 to 1.71	0.569			

BMI, body mass index; CI, confidence intervals; FI, food insecurity; MD, Mediterranean diet; OR, odds ratio; ^†^ based on the MedDietScore (continuous variable) [38]. Note: the dependent variable includes being food-insecure vs. food-secure (reference group).

## Data Availability

Data are available upon request to M.G.G.
